# Beyond BMI: The “Metabolically healthy obese” phenotype & its association with clinical/subclinical cardiovascular disease and all-cause mortality -- a systematic review

**DOI:** 10.1186/1471-2458-14-14

**Published:** 2014-01-08

**Authors:** Lara L Roberson, Ehimen C Aneni, Wasim Maziak, Arthur Agatston, Theodore Feldman, Maribeth Rouseff, Thinh Tran, Michael J Blaha, Raul D Santos, Andrei Sposito, Mouaz H Al-Mallah, Ron Blankstein, Matthew J Budoff, Khurram Nasir

**Affiliations:** 1 Center for Prevention and Wellness Research, Baptist Health Medical Group, Michigan Ave Suite 500, Miami Beach, Florida; 2 Baptist Health South Florida, Miami, Florida; 3 Department of Epidemiology, Robert Stempel College of Public Health, Florida International University, Miami, Florida; 4 The Johns Hopkins Ciccarone Center for the Prevention of Heart Disease, Baltimore, Maryland; 5 Lipid Clinic Heart Institute, University of Sao Paulo Medical School Hospital, Sao Paulo, Brazil; 6 Department of Cardiology, Faculty of Medical Sciences, State University of Campinas (Unicamp), Campinas, São Paulo, Brazil; 7 King Abdul Aziz Cardiac Center, Riyadh, Saudi Arabia; 8 Brigham and Women’s Hospital, Harvard School of Medicine, Boston, Massachusetts, USA; 9 Los Angeles Biomedical Research Institute, Torrance, California, USA; 10 Department of Medicine, Herbert Wertheim College of Medicine, Florida International University, Miami, Florida

## Abstract

**Background:**

A subgroup has emerged within the obese that do not display the typical metabolic disorders associated with obesity and are hypothesized to have lower risk of complications. The purpose of this review was to analyze the literature which has examined the burden of cardiovascular disease (CVD) and all-cause mortality in the metabolically healthy obese (MHO) population.

**Methods:**

Pubmed, Cochrane Library, and Web of Science were searched from their inception until December 2012. Studies were included which clearly defined the MHO group (using either insulin sensitivity and/or components of metabolic syndrome AND obesity) and its association with either all cause mortality, CVD mortality, incident CVD, and/or subclinical CVD.

**Results:**

A total of 20 studies were identified; 15 cohort and 5 cross-sectional. Eight studies used the NCEP Adult Treatment Panel III definition of metabolic syndrome to define “metabolically healthy”, while another nine used insulin resistance. Seven studies assessed all-cause mortality, seven assessed CVD mortality, and nine assessed incident CVD. MHO was found to be significantly associated with all-cause mortality in two studies (30%), CVD mortality in one study (14%), and incident CVD in three studies (33%). Of the six studies which examined subclinical disease, four (67%) showed significantly higher mean common carotid artery intima media thickness (CCA-IMT), coronary artery calcium (CAC), or other subclinical CVD markers in the MHO as compared to their MHNW counterparts.

**Conclusions:**

MHO is an important, emerging phenotype with a CVD risk between healthy, normal weight and unhealthy, obese individuals. Successful work towards a universally accepted definition of MHO would improve (and simplify) future studies and aid inter-study comparisons. Usefulness of a definition inclusive of insulin sensitivity and stricter criteria for metabolic syndrome components as well as the potential addition of markers of fatty liver and inflammation should be explored. Clinicians should be hesitant to reassure patients that the metabolically benign phenotype is safe, as increased risk cardiovascular disease and death have been shown.

## Background

It is well established that obesity continues to rise in prevalence, nearly doubling between the years of 1980 and 2008 worldwide [1]. In the United States between 2009–2010, the rate of obesity was 35.7% in adults and 17% in children [2], which translates into about 78 million adults and 12.5 million children. In literature review, obesity has been shown to be predictive of all-cause, cardiovascular disease (CVD), and cancer mortality [3,4]. Economically, obesity is costly due to its direct association with morbidity and mortality, with the medical costs of treating obesity and its sequelae estimated to be $147 billion in 2009 [5]. From a clinical perspective, the question remains how to best identify those who are at the lowest risk of developing these obesity-related complications, and monitor them in a cost effective way, while allocating the most resources to those in the highest risk group.

A subgroup has been identified within the obese population, who do not display the typical metabolic disorders associated with obesity and are hypothesized to have lower risk of obesity-related complications. Metabolically healthy obesity (MHO), as it will be referred to in this review, has been previously defined as a subgroup of obese individuals who do not have insulin resistance, lipid disorders, or hypertension [6]. Multiple studies indicate 10-25% of the obese can be categorized as MHO [6,7]. Wildman et al. used NHANES, a nationally representative sample of adults living in the US, to examine the MHO phenotype and found a prevalence of 32% among obese adults over the age of 20 [8].

In this review, we aim to evaluate the MHO phenotype in context of cardiovascular disease risk and all-cause mortality. By conducting a systematic literature search which selected studies assessing the association of the MHO phenotype with all-cause mortality, CVD outcomes and subclinical CVD, we sought to summarize the risks in this group, and discuss the challenges faced by studies in examining this unique population.

## Methods

A systematic literature search was conducted using Pubmed, Web of Science, and Cochrane Library. In Pubmed, the following medical subject headings (MESH) and free text terms were used: (“metabolically healthy” OR “metabolically normal” OR “metabolically benign” OR “insulin sensitive”) AND (“obese” OR “overweight”). The same text word search was used in Web of Science and Cochrane Library as in Pubmed. No restrictions were used for publication status or date, but studies had to be peer-reviewed to be selected. Libraries were searched until December 2012. Studies were selected if they were written in English, included human subjects over the age of 18, defined a metabolically healthy obese/overweight group and contained either a measure of subclinical cardiovascular disease or a hard outcome such as death or cardiovascular disease. Studies were excluded if they failed to include a comparison group to the MHO, the abstract and full text was unavailable, or were reviews, letters, and case-reports. Studies were also excluded if they failed to define metabolically healthy using either insulin sensitivity or components of metabolic syndrome or failed to define obesity using BMI, body fat percentage, or waist circumference. Two reviewers independently selected articles, and then jointly agreed on the final studies to be included.

## Results

### Eligible studies

**Figure 1:**
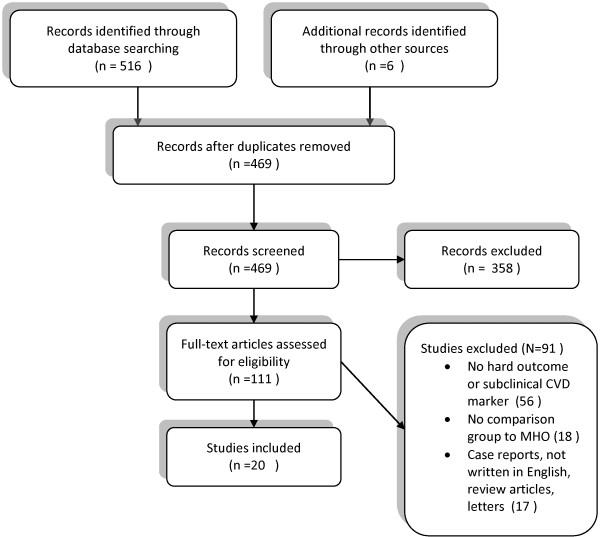
Search flow chart.

Our search identified a total of 340 studies from Pubmed, 156 from Web of Science, and 20 from Cochrane library. Six more studies were identified by searching the reference lists of all full-text papers included from the initial database search. Twenty articles met the final inclusion criteria (Figure 1).

### Cohort studies

A total of 14 cohort studies were identified which examined the MHO phenotype and mortality or cardiovascular outcomes [9-22] (Tables 1 and 2). These studies reported an average follow-up time of 11 years. In total, the 14 studies examined 359,137 participants, 9,865 fitting the MHO phenotype, or 2.7% of the total population studied. One study examined the MHO phenotype and progression of subclinical cardiovascular disease at three-year follow-up [23]. The majority of studies examined risks in Caucasian subjects or subjects of European descent (*n* = 12). One study used an Iranian cohort [12] and one study did not report ethnicity [13]. On average, participants were over the age of 50 (*n* = 9), while a small segment of studies reported younger average age (*n* = 4), or did not report age [13]. One cohort study examined the MHO phenotype and subclinical CVD [23] (Tables 3 and 4).

**Table 1:** **MHO definition; *cohort studies***

**Study**	**Definition of metabolically healthy obese (MHO)**	**Definition of metabolically healthy, normal weight (MHNW)**
Katzmarzyk [19]	• <3 MetS	• ≤ 2 MetS criteria, normal weight (BMI 18.5-24.9 kg/m^2^)
	• BMI ≥30 kg/m^2^	
	• Diabetics included	
St-Pierre [17]	• ≤2 insulin resistance syndrome^A^ (IRS) criteria	• ≤2 IRS^A^ criteria, normal weight (BMI <25 kg/m^2^)
	• BMI ≥ 30 kg/m^2^	
	• Diabetics excluded	
Meigs [14]	• < 3 MetS criteria and/or IS by HOMA-IR < 75th percentile	• <3 MetS criteria, normal weight (BMI < 25 kg/m^2^) and/or IS, normal weight (BMI < 25 kg/m^2^)
	• BMI ≥ 30 kg/m^2^	
	• Diabetics excluded	
Daly [20]	• <3 MetS^B^	• <3 MetS^B^ , normal weight (BMI <25 kg/m^2^)
	• BMI > 30 kg/m^2^	
	• Diabetics included	
Song [21]	• < 3 MetS^*C*^ criteria	• <3 MetS^*C*^ , normal weight (BMI <25 kg/m^2^)
	• BMI ≥30 kg/m^2^	
	• Diabetics included	
Kuk [13]	• ≤1 MetS criteria and/or IS by HOMA < 2.5	• ≤1 MetS criteria, normal weight (BMI < 25 kg/m^2^) and/or IS, normal weight (BMI < 25 kg/m^2^)
	• BMI ≥ 30 kg/m^2^	
	• Diabetics included	
Arnlov [9]	• <3 MetS^D^ criteria and/or IS by HOMA-IR <75th percentile	• <3 MetS^D^ criteria, normal weight (BMI < 25 kg/m^2^) and/or IS, normal weight (BMI < 25 kg/m^2^)
	• BMI > 30 kg/m^2^	
	• Diabetics excluded	
Calori [10]	•IS by HOMA-IR < 2.5	• IS, not obese (BMI < 30 kg/m^2^)
	• BMI ≥ 30 kg/m^2^	
	• Diabetics included	
Voulgari [18]	• < 3 MetS criteria	• <3 MetS criteria, normal weight (BMI <24.9 kg/m^2^)
	• BMI ≥ 30 kg/m^2^	
	• Diabetics excluded	
Hosseinpanah [12]	• <3 MetS^E^ criteria	• <3 MetS^E^ criteria, normal weight (BMI 18.5-24.9 kg/m^2^)
	• BMI ≥30 kg/m^2^	
	• Diabetics included	
Bobbioni-Harsch [23]	• 0 MetS criteria	• 0 MetS criteria, normal weight (BMI < 25 kg/m^2^)
	• BMI ≥25 kg/m^2^	
Ogorodnikova [15]	• ≤ 2 MetS^C^ criteria; ≤1 of any MetS^C^ criteria, IR by HOMA-IR > 75th percentile of all participants, or systemic inflammation by WBC >75th percentile for all participants; or IS by HOMA-IR ≤25th percentile among non-diabetic obese, sex-specific	• ≤ 2 MetS^C^ criteria, normal weight (BMI 18.5-24.9 kg/m^2^) or ≤ 1 MetS^C^ criteria, IR by HOMA-IR > 75th percentile of all participants, or systemic inflammation by WBC >75th percentile for all participants, normal weight (BMI 18.5-24.9 kg/m^2^) or IS, normal weight (BMI 18.5-24.9 kg/m^2^)
	• BMI ≥ 30 kg/m^2^	
	• Diabetics included	
Hamer [11]	• <2 MetS^F^ criteria (including CRP *≥3.0 mg/L)*	• <2 MetS^F^ criteria, not obese (BMI 18–29.9 kg/m^2^) or <2 MetS^F^ criteria, normal waist (≤102 cm in men, ≤88 cm in women)
	• BMI ≥30 kg/m^2^ or waist circumference >102 cm men, >88 cm women	
	• Diabetics included	
Bo [22]	• < IS by HOMA < 2.5 AND <3 MetS^G^ criteria	• IS, normal weight (BMI <25 kg/m^2^)
	• BMI > 30 kg/m^2^	
	• Diabetics included	
Ortega [16]	• ≤1 MetS^C^ criteria	• ≤1 MetS^C^ criteria, normal weight (BMI 18.5-24.9 kg/m^2^) or • ≤1 MetS^C^ criteria, normal body fat (<25% men/<30% women)
	• Body Fat% ≥ 25/30 (M/F) ORBMI ≥ 30 kg/m^2^	
	• Diabetics included	

^A^*TG ≥150 mg/dL, HDL <50 mg/dL, ≥54.5% of LDL particles with diameter <255A, Apo lipoprotein B level ≥1.36 g/L, fasting insulin level ≥ 85.2 pmol/L, blood pressure ≥ 135/85 mmHg,* CRP ≥3.0 mg/L; ^*B*^*TG and waist circumference excluded, added BMI > 30 kg/m2 into criteria;*^*C*^*Waist circumference excluded*; ^D^*Waist circumference excluded, added BMI ≥ 29.4 kg/m2 cut-point into criteria*; ^E^*Waist circumference ≥89 cm for men and ≥91 cm for women, blood pressure ≥ 140/85 mmHg*; ^F^*TG excluded, added* CRP ≥ 3 mg/L *into criteria*; ^*G*^*Waist circumference ≥94 cm for men and ≥80 cm for women, fasting plasma glucose ≥ 100 mg/dl or use of hypoglycemic therapy.*

**Table 2:** Results; cohort studies

**Study**	**Subjects (age , male)**	**Median follow-up time (yrs)**	**Outcome**	**Results**
Katzmarzyk [19]	N = 19,173	10	All-cause mortality CVD mortality	*MHO* = All cause: 25 (3%); CVD: 12 (1%); *MHNW* = All-cause: 160 (2%); CVD: 38 (1%);
	MHO = 1,019 (43, 100%)			•Unadjusted HR for MHO vs. MHNW = NR (All-cause); NR (CVD)
	MHNW = 7,153 (41, 100%)			•Adjusted^A^ HR for MHO vs. MHNW = 0.88 (0.57-1.36) [All-cause]; 1.59 (0.81-3.12) [CVD]
St-Pierre [17]	N = 1,824	13	Incident CVD	Total = 284 (16%); *MHO* = NR; *MHNW* = NR
	MHO = 54 (NR, 100%)			•Unadjusted HR for MHO vs. MHNW = NR
	MHNW = 512 (NR, 100%)			•Adjusted^B^ HR for MHO vs. MHNW = 1.53 (0.79-3.00)
Meigs [14]	N = 2,902	11	Incident CVD	*MHO* = CVD: 19 (8%); *MHNW* = CVD: 19 (5%);
	MHO = 236 (52, 51%)			•Unadjusted RR for MHO vs. MHNW = NR
	MHNW = 981 (52, 27%)			• Adjusted^C^ RR for MHO (MetS) vs. MHNW = 1.48 (0.87-2.55)[CVD]
				•Adjusted^C^ RR for MHO (IS) vs. MHNW = 1.42 (0.87-2.33) [CVD]
Daly [20]	N = 8,397 (60, 87%)	4	CVD mortality	*MHO* = NR (3%); *MHNW* = NR (2%)
	MHO = 839 (NR,NR)			•Unadjusted RR for MHO vs. MHNW = 1.42 (0.76-2.63)
	MHNW = NR			•Adjusted RR for MHO vs. MHNW = NR
Song [21]	N = 25,626	10	Incident CVD	*MHO* = CVD: 77 (3%); *MHNW* = CVD: 278 (2%)
	MHO = 2,925 (54, 0%)			•Unadjusted RR for MHO vs. MHNW = NR
	MHNW = 12,943 (54, 0%)			•Adjusted^D^ RR for MHO vs. MHNW = 1.05 (0.66-1.66)
				•Adjusted^E^ RR for MHO vs. MHNW = 1.07 (0.68-1.70)
Kuk [13]	N = 6,011	9	All-cause mortality	Total = 292 (5%); *MHO* = NR; *MHNW* = NR
	MHO = 78 (NR, NR)			•Unadjusted RR for MHO (MetS) vs. MHNW = NR
	MHNW = 1,461 (NR, NR)			•Adjusted^F^ RR for MHO (MetS) vs. MHNW = **2.80 (1.18-6.65)**
				•Unadjusted RR for MHO (IS) vs. MHNW = NR
				•Adjusted^F^ RR for MHO (IS) vs. MHNW = **2.58 (1.00-6.65)**
Arnlov [9]	N = 1,758	30	All-cause mortality	*MHO* = All-cause: 18 (60%); CVD mortality: 5 (17%) *MHNW* = All-cause: 391 (44%); CVD mortality: 155 (17%)
	MHO = 30 (50, 100%)		CVD mortality	•Unadjusted RR for MHO (MetS) vs. MHNW = NR
	MHNW = 891 (50, 100%)		Incident CVD	• Adjusted^G^ RR for MHO (MetS) vs. MHNW = **1.65 (1.03-2.66)** [All-cause]; 1.20 (0.49-2.93) [CVD mortality]; **1.95 (1.14-3.34)** [Incident CVD]
				• Adjusted^G^ RR for MHO (IS) vs. MHNW = **2.04 (1.25-3.32)** [All-cause]; 1.80 (0.79-4.08) [CVD mortality]; **1.91 (1.07-3.41)** [Incident CVD]
Calori [10]	N = 2,011	15	All-cause mortality	*MHO =* All-cause: 7 (16%); CVD: 2 (5%) *MHNW* = All-cause: 141 (20%); CVD: 58 (8%)
	MHO = 43 (55, 28%)		CVD mortality	•Unadjusted HR for MHO vs. MHNW = NR
	MHNW = 708 (55, 45%)			•Adjusted^H^ HR for MHO vs. MHNW = 0.99 (0.46-2.11) [All-cause]; 0.73 (0.18-3.00) [CVD]
Voulgari [18]	N = 550 (60, NR)		Incident heart failure	*MHO* = 43 (9%); *MHNW* = 17 (16%)
	MHO = 96 (NR, NR)	6		•Unadjusted HR for MHO vs. MHNW = 0.26 (NR)
	MHNW = 109 (NR, NR)			•Adjusted^I^ HR for MHO vs. MHNW = 0.41 (0.10-1.31)
Hosseinpanah [12]	N = 6,215	8	Incident CVD	*MHO* = 13 (3%); *MHNW* = 64 (4%)
	MHO = 408 (45, 20%)			•Unadjusted HR for MHO vs. MHNW = NR
	MHNW = 1,555 (45, 57%)			•Adjusted^J^ HR for MHO vs. MHNW = 1.07 (0.59-1.96)
Bobbioni Harsch [23]	N = 436	3	CCA-IMT	Male Subjects *Mean CCA-IMT at Baseline*: MHO = 0.63 (0.07) vs. MHNW = 0.59 (0.08) p = NS
	MHO = 65 (45, 42%)			*Mean CCA-IMT at 3 years:* MHO = 0.62 (0.10) vs. MHNW = 0.60 (0.07) p = NS
	MHNW = 194 (43, 23%)			Female Subjects *Mean CCA-IMT at Baseline*: MHO = 0.57 (0.06) vs. MHNW = 0.56 (0.06) p = NS
				*Mean CCA-IMT at 3 years:* MHO = 0.61 (0.08) vs. MHNW = 0.58 (0.06) **p < 0.05**
Ogorodnikova [15]	N = 17,544	12	Incident CVD	Total = 2,439 (14%); *MHO* = NR; *MHNW* = NR
	MHO = 1,167 (56, 32%)			•Unadjusted HR for MHO vs. MHNW = NR
	MHNW = 4,036 (58, 33%)			•Adjusted^K^ HR for MHO (using various definitions) vs. MHNW = **1.30 (1.03-1.66)** [ATP III definition]; 1.17 (0.87-1.57) [ATP-III Expanded]; **1.52 (1.19-1.95)** [IS definition]
Hamer [11]	N = 22,203	7	All-cause mortality	*MHO =* All-cause: 38 (3%); CVD: 18 (2%);
	MHO = 1,160 (51.3, 53%)		CVD mortality	*MHNW* = All-cause: 777 (6%); CVD: 225 (2%)
	MHNW = 12,716 (51.9, 45%)			•Unadjusted HR for MHO vs. MHNW = NR
				•Adjusted^L^ HR for MHO v. MHNW = 0.91 (0.64-1.29) [All-cause]; 1.26 (0.74-2.13) [CVD]
Bo [22]	N = 1,658	9	All-cause mortality	*MHO =* All-cause: 15 (21%); CVD mortality: 8 (11%); Incident CVD: NR (8%)
	MHO = 72 (55, 33%)		CVD mortality	*MHNW* = All-cause: 28 (5%); CVD mortality:7 (1%);Incident CVD: NR (3%)
	MHNW = 540 (54, 38%)		Incident CVD	•Unadjusted HR for MHO vs. MHNW = NR
				•Adjusted^M^ HR for MHO vs. MHNW = 1.36 (0.64-2.08) [All-cause]; **2.48(1.35-3.61) **[CVD mortality]; **2.76 (1.05-7.28)** [Incident CVD]
Ortega [16]	N = 43,265	14	All-cause mortality	*MHO =* All-cause: 52 (3%); CVD mortality: 17 (1%); Incident CVD: 30 (6%)
	MHO = 1,738 (NR, 80%)		CVD mortality	*MHNW* = All-cause: 449 (3%); CVD mortality: 98 (1%); Incident CVD: 261 (4%)
	MHNW = 16,002 (NR,NR)		Incident CVD	•Unadjusted HR for MHO(referent) vs. MHNW = NR
				•Adjusted^N^ HR for MHO(referent) vs. MHNW = 0.91(0.67-1.24) [All-cause]; 0.73 (0.42-1.28) [ CVD mortality]; 0.78 (0.52-1.18) [Incident CVD]

^A^Adjusted for age, year of exam, smoking, alcohol use, possible existence of CVD, parental history or premature CVD, cardio-respiratory fitness; ^B^Adjusted for age, smoking, and medication use at baseline; ^C^Adjusted for age, sex, LDL, smoking; ^D^Adjusted for age, treatment assignment, smoking, exercise, alcohol use, total calorie intake, postmenopausal hormone use, multivitamin use, parental history of myocardial infarction before 60 years, BMI; ^E^Adjusted for age, treatment assignment, smoking, exercise, alcohol use, total calorie intake, postmenopausal hormone use, multivitamin use, parental history of myocardial infarction before 60 years, CRP; ^F^Adjusted for age, sex, income, smoking, ethnicity, alcohol use; ^G^Adjusted for age, smoking, and LDL; ^H^Adjusted for age, sex; ^I^Adjusted for age, sex, impaired glucose tolerance, dyslipidemia, hypertension, smoking, physical inactivity, left ventricular hypertrophy, and function on echocardiography; ^J^Adjusted for age, gender, exercise, smoking, family history of premature CAD, high total cholesterol; ^K^Adjusted for age, gender, race, smoking, education, alcohol use, and study cohort; ^L^Adjusted for age, sex, smoking physical activity, socioeconomic group, and BMI; ^M^Adjusted for age, sex, fiber intake, exercise, waist circumference; ^N^Adjusted for age, sex, examination year, smoking, alcohol, parental history of CVD, fitness.

**Table 3:** MHO Definition; Cross Sectional Studies

**Study**	**Definition of metabolically healthy obese (MHO)**	**Definition of metabolically healthy, normal weight (MHNW)**
Marini [25]	• IS by OGTT & euglycemic clamp ≥75th percentile of all obese participants	• Not obese (BMI < 27 kg/mg2)
	• BMI >30	
	• Diabetics included	
Stefan [26]	• IS by OGTT ≥75th percentile of all obese participants	• IS, normal weight (BMI < 25 kg/m2)
	• BMI ≥ 30	
	• Diabetics included	
Irace [29]	• <3 MetS criteria	• <3 MetS, normal weight (BMI 18.5-25 kg/m2)
	• BMI > 29.9	
	• Diabetics included	
Khan [27]	• <3 MetS^A^ Criteria (including CRP *≥3.0 mg/L)*	• <3 MetS^A^ criteria, normal weight (BMI < 25 kg/m2)
	• BMI ≥ 25	
	• Diabetics included	
Park [28]	• 0 MetS^B^ criteria	• 0 MetS^B^ criteria, normal weight (BMI <23 kg/m2)
	• BMI ≥ 25	
	• Diabetics included	

^A^*Waist circumference excluded, added* CRP ≥3.0 mg/L; ^B^W*aist circumference ≥90 cm for men and ≥80 cm for women, fasting glucose at least 100 mg/dL.*

**Table 4:** Results; Cross Sectional Studies

**Study**	**Subjects (Age, Male)**	**Outcome**	**Results**
Marini [25]	N = 153	CCA-IMT	*Mean CCA-IMT*: MHO = 0.79 (0.08) vs. MHNW = 0.61 (0.11) **p < 0.001**
	MHO = NR (35, 0%)		
	MHNW = 73 (34, 0%)		
Stefan [26]	N = 314	CCA-IMT	*Mean CCA-IMT:* MHO = 0.54 (0.02) vs. MHNW = 0.51 (0.02) p = NS
	MHO = 31 (47, 39%)		
	MHNW = 54 (45, 17%)		
Irace [29]	N = 1842 (30–80 years old, 55%) MHO = NR	CCA-IMT	*Mean CCA-IMT:* MHO = NR vs. MHNW = NR
	MHNW = NR		• Adjusted^A^ OR of CCA-IMT MetS: 1.42 (1.10-1.83) **p = 0.01**BMI: p = NS
Khan [27]	N = 475	CCA-IMT	*Mean CCA- IMT:* MHO = 0.68 (0.09) vs. MHNW =0.64 (0.08) **p < 0.001**
	MHO = 260 (51, 0%)	aPWV	*Mean aPWV*: MHO = 809.9 (182.3) vs. MHNW =731 (176.4) **p < 0.001**
	MHNW = 145 (51, 0%)	CAC	*Frequency (%) with increased CAC:* MHO = 53 (20%) vs. MHNW =13 (9%) **p < 0.001**
		AC	*Frequency (%) with increased AC*: MHO = 130 (50%) vs. MHNW =47 (32%) **p < 0.001**
			•Adjusted^B^ OR's of CAC and AC associated with MHO vs. MHNW CAC: 2.38 (1.20,4.70) **p = 0.01**; AC: 2.37 (1.49.3.80) **p < 0.001**
			•Adjusted^B^ regression coefficients associated with MHO vs. MHNW CCA-IMT: 0.034 **p < 0.001**; aPWV: 59.7 **p = 0.001**
Park [28]	N = 2540 MHO = 71 (52, 45%)	CCA-IMT	*Mean CCA IMT:* MHO = 0.72 (0.06) vs. MHNW = 0.70 (0.06) p = NS
	MHNW = 286 (52, 52%)	aPWV	*Mean aPWV:* MHO = 12.8 (1.1) vs. MHNW = 12.9 (1.6) p = NS
		LVMI	*Mean LVMI*: MHO = 42.4 (7.4) vs. MHNW = 35.4 (6.6) **p < 0.01**
		E/A ratio	*Mean E/A ratio*: MHO = 1.17 (0.34) vs. MHNW = 1.34 (0.41) **p < 0.05**

^A^Adjusted for age, sex, smoking, metabolic syndrome, BMI, LDL; ^B^Adjusted for age, site of recruitment, education, race, smoking; ^C^Adjusted for age, sex, heart rate, CRP, medication for hypertension, treatment for diabetes mellitus.

#### *i.* Definitions of MHO

Studies were examined in reference to their definition of MHO (Table 1). In 13 of the studies, obesity was defined as BMI ≥ 30 kg/m^2^. The remaining study defined obesity as either BMI ≥ 30 kg/m^2^ or body fat > 25% in men and 30% in women [16]. Three studies included measures of inflammation (either C-Reactive protein [CRP] or white blood cell [WBC] count) in the definition of MHO [11,15,17]. Two studies defined “metabolically healthy” as absence of metabolic syndrome (MetS) based on the NCEP ATP III definition of MetS without any modification [18,19]. Five studies defined “metabolically healthy” as absence of MetS with study specific modifications to the MetS criteria [11,12,16,20,21]. One study used only insulin sensitivity (IS) to define “metabolically healthy” [10]. Two studies created a unique definition using a combination of insulin sensitivity (IS) and MetS [17,23]. Lastly, four studies examined both the IS and MetS definitions of “metabolically healthy” within the same study [9,13-15].

#### ii. Outcomes measured

a. *All-cause mortality.* Seven studies reported all-cause mortality [9-11,13,16,19,23]. MHO was found to be significantly associated with all-cause mortality in two studies (30% of studies). Five studies used metabolically healthy, normal weight (MHNW) as the comparison group [9,13,16,19,23] of which the two significant studies were included. Two used metabolically normal, “not obese, BMI <30 kg/m^2^” [10,11] as the comparison and both reported null results. Finally, four studies controlled for physical fitness (either reported exercise or measured cardio-respiratory fitness) of which all reported null results [11,16,19,24].

b. *CVD mortality.* Seven studies separately analyzed CVD mortality [9-11,16,19,20,23]. Five studies showed no statistically significant association between MHO and CVD mortality, but pointed towards higher CVD mortality in the MHO [9,11,16,19,20]. Only one study (14% of reviewed studies) found the metabolically healthy obese to have significantly higher risk of CVD mortality [23]. The studies using “metabolically normal, not obese” rather than “normal weight” as their comparison group found no association between MHO and CVD mortality [10,11].

c. *Incident CVD.* Nine studies examined incident CVD events [9,12,14-19,21,23]. Incident CVD was defined as stroke, coronary heart disease (CHD), and/or heart failure. Three studies (33%) reported significant increase in risk of CVD events among the MHO [9,15,23]. Five studies showed no statistically significant association between incident CVD and MHO, but consistently pointed towards an incidence of CVD in the MHO approximately 1.5 times that of the comparison group [11,12,14,17,21]. The single study which was non-significant and did not point towards an increase in risk had a follow up of 6 years, small sample size of 550, and measured only incident heart failure [18].

d. *Subclinical measures of CVD.* Bobbioni-Harsch et al. examined the three year progression of common carotid intima medial thickness (CCA-IMT), a measure of arterial wall thickness and indicator of atherosclerotic disease progression, in a cohort of 376 metabolically healthy adults aged 30 to 60 years [23] (Tables 1 and 2). At baseline and three years, cardio-metabolic risk factors were measured. Participants were grouped by weight class and according to their metabolic status at follow up. They were considered at-risk if they had developed one or more cardio-metabolic risk factors. At baseline, there was no significant difference in mean CCA-IMT between weight classes. Among the metabolically normal at follow-up, mean CCA-IMT was significantly higher in obese women as compared to normal body weight women. In regression, BMI was significantly associated with the occurrence of ≥1 cardio-metabolic risk factors in all subgroups at follow-up. The incidence of one or more cardio-metabolic risk factors was 57.2% in overweight and obese participants who were metabolically normal at baseline, compared with 31.7% of normal weight participants. Also, they found independent of metabolic abnormalities, the CCA-IMT was thicker in the obese and overweight, than in the normal weight.

#### iii.*Other considerations*

a. *Inflammation*. A total of 5 of the 14 included studies reported any measure of inflammation. Four studies documented C-reactive protein (CRP) levels [11,17,21,22]. One study used white blood cell count > 75^th^ percentile as a marker of inflammation [15]. Hamer, Ogorodokniva and St Pierre included vascular inflammation in the definition of metabolic health [11,15,17]. Only one study adjusted for the presence of vascular inflammation in predicting CVD [21]. None of these studies reported the association of vascular inflammation with CVD outcomes.

b. *Risk-reducing behaviors.* Eight of 14 studies included measures of exercise or fitness [11-13,16,18,19,21,23]. One study measured cardio-respiratory fitness by treadmill test and found that increased physical activity offset the increased CVD mortality seen in the MHO [19]. Seven studies utilized self-reported measures of physical activity and found that the MHO had higher physical activity levels as compared to the MUHO. However, these studies did not assess the effect of physical activity alone on CVD outcomes in the metabolically healthy obese. Only three studies documented dietary behaviors in the study populations [13,21,23]. All three utilized self reported methods. Out of these, one study found that in subjects with BMI < 30, insulin sensitive individuals had significantly higher mean daily fiber consumption as compared to insulin resistant subjects. No significant difference was found in fiber consumption between insulin sensitive or resistant subjects with BMI > 30 [23].

### Cross sectional studies

A total of five cross sectional studies met inclusion criteria, [25-29] (Tables 3 and 4). In total 5,234 participants were examined for prevalence of subclinical CVD in relation to the MHO phenotype.

#### *i.* Definitions of MHO

All studies included diabetics. Two studies defined obese/overweight as BMI ≥ 25 kg/m^2^[27,28]. The remaining studies used BMI > or ≥ 30 kg/m^2^. Two studies defined MHO using IS [25,26]. Of the remaining three studies, one used the standard NCEP ATP III definition of MetS [29], and the other two used a modified version of the MetS definition [27,28]. One study included CRP < 3 mg/L in the definition of “metabolically healthy” [27].

#### ii Outcomes measured

a. *Common carotid artery-intima medial thickness.* All five of the studies measured CCA-IMT as a marker of atherosclerosis. Of the four studies reporting mean difference in CCA-IMT between MHO and MHNW individuals [25-28], two reported significantly higher levels in the MHO [25,27]. However, in the two studies that did not attain statistical significance, the mean CCA-IMT tended to be higher in the MHO group as compared to the MHNW group [26,28]. The final study determined that carotid atherosclerosis was similar among normal weight, overweight, and obese participants when stratified by presence or absence of MetS,

b. *Other subclinical measures of CVD.* One study assessed coronary artery calcium (CAC), which is an early indicator of atherosclerotic disease measuring the degree of calcification in the coronary arteries [27]. Khan et al. reported higher mean levels of CAC and increased frequency of higher CAC in the metabolically healthy obese as compared to controls. One study assessed liver fat, and found that the frequency of MHO participants with fatty liver was significantly higher than that of normal weight, but lower than metabolically unhealthy obese participants [26]. Finally, one study also examined multiple measures of cardiovascular changes including left ventricular mass index(LVMI), mitral E/A ratio, E/Ea ratio, TDI and Ea velocity [28]. Park et al. found the MHO had significantly increased LVMI and decreased mitral E/A ratio as compared to the metabolically normal, normal weight participants; indicating changes in heart structure and function in this group [28].

#### iii.*Other considerations*

a. *Inflammation.* CRP was measured in two studies [27,28] neither of which reported the association of CRP and CVD outcomes in the context of metabolic health and obesity. Khan et al. included vascular inflammation (VI) in the definition of metabolic syndrome [27]. Park adjusted for VI in predicting subclinical CVD outcomes studied [28].

## Discussion

The purpose of the present study was to review the literature that has examined the relationship between the metabolically healthy obese phenotype and cardiovascular disease. The most common definition of MHO used was having a BMI ≥ 30 kg/m^2^ and less than 3 MetS criteria (8 studies). Nine studies also included insulin sensitivity in the definition of MHO. The most commonly used comparison group was MHNW defined as having a BMI < 25 kg/m^2^ and meeting < 3 MetS criteria (9 studies). Of the fourteen studies identified examining CVD outcomes or all-cause mortality, most were not able to demonstrate a significant association between MHO and increased risk of CVD and mortality. However, a trend towards a slight increase in risk in all but one study was observed, which was supported by increased subclinical disease burden observed in the cross-sectional studies. In the six studies measuring subclinical CVD, the MHO phenotype was associated with increased subclinical CVD burden in four, and this association achieved statistical significance. It appears that MHO individuals have a slightly increased CVD risk as compared to their normal weight counterparts, but the results are mixed as can be seen from the studies reviewed here. It is difficult to determine whether the mixed findings are a result of methodological issues (such as variable follow-up duration, small sample size, non-standardized comparison groups, outcome measurement, etc.) or due to a truly weak effect. Therefore, there is a pressing need for establishing a streamlined, central definition of “metabolically healthy obese” in future studies.

### Defining “metabolically healthy obese”

As mentioned previously, the definitions of MHO are quite heterogeneous and make quantitative synthesis of the reviewed studies difficult. One of the first to identify and review this phenotype in 2001, was Sims [30], who highlighted visceral adiposity and insulin resistance as key determinants of MHO. In the literature analyzed for this review, the most commonly applied definition was the absence or presence of MetS in conjunction with obesity (measured via BMI). IR was often ignored and replaced with fasting glucose. BMI, though a useful tool for estimating body fatness, has limitations in that it does not discriminate between lean muscle and fat, or fat distribution. Waist circumference, however, provides an estimate of visceral adiposity which many studies have shown to be significantly associated with IR, type 2 diabetes, and cardiovascular events [31]. Fasting glucose, used in the diagnosis of MetS, has limitations in that in only provides a snap-shot of glucose regulation [32]. Ideally, IR should be measured using the gold standard diagnostic test, the hyperinsulinemic euglycemic glucose clamp (HEGC) [33]. However, this procedure is costly, labor intensive for the investigator, and largely uncomfortable for the patient. Less invasive, but still labor intensive tests such as HOMA-IR or the quantitative insulin sensitivity check index (QUICKI) have been shown to produce similar results and may be useful substitutes for HEGC.

Two often-discussed points in MHO are inflammation as measured by C-reactive protein (CRP) and fatty liver or non-alcoholic fatty liver disease (NAFLD). Inflammation has been shown to promote IR [34]. CRP is thought to be the best biomarker of vascular inflammation and has been shown to be predictive of CVD events [34,35]. In the studies reviewed here, 4 studies included CRP in the definition of MHO, but none of them assessed the relationship of VI and CVD risk. Therefore, the usefulness of CRP as part of the definition of MHO, or as a predictor of CVD events in the metabolically healthy obese remains uncertain in the studies reviewed here. It is difficult to draw conclusions regarding the usefulness of including VI in the definition of metabolic health or its role as a predictor of CVD from the studies included in this review. Absence of NAFLD has also been explored as a potential identifier of MHO. Fatty liver has been implicated as contributing to IR, and has been associated with increased risk of incident cardiovascular disease even when controlling for metabolic syndrome and obesity [36,37]. Messier et al. found that the MHO had lower liver fat content than their “at-risk” obese equivalents [38]. One study reviewed examined fatty liver in the MHO and found them to have a prevalence of NAFLD similar to overweight participants and greater than those in the normal weight category [26]. Going forward, liver fat could prove a useful tool to diagnose and assess metabolic health of the obese, especially when other metabolic abnormalities are absent. NAFLD can be assessed non-invasively using ultrasound, and provides an early diagnostic marker for cardiovascular disease risk. More long-term studies are needed to confirm its usefulness in the MHO population.

Future studies would be well served to use an increasingly strict definition of MHO which requires measures of the most significant features (visceral adiposity and IR) while still including information on blood pressure, fasting glucose, cholesterol, triglycerides, liver fat, and inflammation. To show a greater difference in risk, defining MHO as having none of the components may have greater utility. St-Pierre et al. found the relative risk of ischemic heart disease increased linearly with increasing number of metabolic abnormalities [17]. No study reviewed used 0 MetS components to define MHO. However, among the three cohort studies which used ≤1 MetS criteria in the definition of MHO, only one showed statistically significant difference in the MHO as compared to MHNW [13].

Equally important as defining MHO is the need to determine appropriate comparison groups. In this review, we chose to look at only the comparison between MHO and the “healthiest” group. This was defined as the metabolically healthy, normal weight participants (BMI < 25 kg/m^2^) in most studies; however, some studies chose to use only normal weight individuals with no report of metabolic health, or included overweight individuals in the comparison group. Using these parameters, “unhealthy” traits may have leaked into the “healthiest” groups, leading to crossover bias in analysis which would make true differences between these groups more difficult to detect. No study has compared CVD risks in the MHO by class of obesity. Soverini et al. examined the prevalence of metabolic syndrome and insulin resistance in morbidly obese patients, and found a small percentage maintained insulin sensitivity even at BMI > 40 kg/m^2^[39]. When comparing MHO to the healthiest, the sickest, or even an intermediary (metabolically unhealthy and normal weight), this review stresses the importance of a well defined comparison group in order to make inferences relevant to the general population.

### Subclinical disease measures

When assessing intermediate risk populations such as the obese, the usefulness of traditional risk factor assessment (such as Framingham) diminishes. Measures of subclinical disease are increasingly important as they provide a view of actual disease progression and more accurate event prediction than traditional CVD risk factors. The most common subclinical disease measures used are CCA-IMT, CAC, ankle-brachial index (ABI), and endothelial function. CCA-IMT was the most commonly studied measure of subclinical disease in the studies reviewed. A majority of the studies documented a significant trend towards increased IMT in MHO versus controls. The rising gold standard for measuring degree of atherosclerosis is CAC. Only one study in our analysis measured CAC and found it was significantly increased in the MHO. No study to date has examined whether the differences in outcome (incident CVD and CVD mortality) can be explained by differences in subclinical CVD. In the studies which examined subclinical disease, all except one were cross-sectional in design, which gives us a snapshot of subclinical disease in the MHO, but does not allow us to examine the progression of disease. The prospective study by Bobbioni-Harsh et al. gave important insights into the development and progression of subclinical disease among the metabolically healthy obese, showing faster progression of subclinical CVD, independent of metabolic abnormalities [23]. Cohort studies allow us to see the time sensitive component of obesity, and the progressive development of cardiovascular risk factors. Future follow-up studies are needed which include multiple measures of subclinical CVD at baseline and follow-up and examine MHO in context of increased burden as compared to their normal weight counterparts.

### Expanding the subject population

Cardiovascular disease prevalence, morbidity, and mortality difference are well established across racial/ethnic and socioeconomic lines [40,41]. Surveillance data from NHANES and BRFSS indicate African Americans experience higher death rates due to both heart disease and stroke [40]. Likewise, Hispanics and Non-Hispanic blacks have higher levels of obesity as compared to Non-Hispanic whites [2]. Of the current studies reviewed, all but one assessed CVD risk in the MHO phenotype in participants of mainly Caucasian or European descent. Surveillance data likewise indicates the prevalence of heart disease, hypertension, and stroke in those persons with incomes below the poverty threshold, is significantly greater than those at or above the poverty threshold [41]. We did not identify any study which examined the prevalence of the MHO phenotype in association with CVD risk in the MHO across racial or socioeconomic lines. Thus, there is an urgent need for longitudinal studies with socioeconomically diverse, multi-ethnic populations in order to correctly identify the CVD risk of the MHO group.

### Mechanisms of MHO

Behavioral and lifestyle factors may play a large role in why a subset of the obese do not present with metabolic abnormalities or present them at a slower pace. However, this is a topic which has not been studied at length and presents mixed findings. Seven of the eight studies measuring physical activity found increased activity in the metabolically healthy obese, when compared to the metabolically unhealthy obese. In addition, several studies found that increased physical activity offset the increased risk of CVD in the MHO. This suggests that all studies examining the MHO phenotype should also assess physical activity, as physical activity appears to nullify the effect of MHO on CVD risk. Hankinson et al. found no difference between diet factors, and physical activity reported between MHO and unhealthy obese participants [42]. However, Messier and colleagues found engaging in physical activity increased the odds of presenting with the MHO phenotype among obese participants [38]. A review by Gill found that increasing physical activity reduced IR, a key factor in defining MHO [43]. While most studies measured physical activity by self report, more accurate results are obtained when physical fitness is directly measured. Ortega et al. measured cardio-respiratory fitness by treadmill test and found MHO participants had significantly higher VO2 max when compared to the metabolically unhealthy obese [16]. Recent research on sedentary behavior, independent of physical activity, has shown that increased sedentary time is associated with increased risk of diabetes, cardiovascular events, and cardiovascular as well as all-cause mortality [44]. This is in agreement with the belief that the natural tendency of the MHO is to progress to MUHO when physical activity is stopped or reduced [45]. To date, no study has examined sedentary time in the MHO phenotype.

Future research should also target genetic studies to illuminate why some obese individuals do not develop or experience delayed development of metabolic syndrome. For example, one study in mice found that extremely obese mice with a mutation in the Brd2 gene are protected from developing type 2 diabetes mellitus [46]. This could provide a clue as to why certain obese individuals do not seem to develop the metabolic abnormalities associated with obesity. However, the search for relevant genes in human models continues. It is likely that a gene-environment interaction is at play, similar to the model suggested for the development of obesity [47]. More follow-up studies are needed to examine the transition between MHO and metabolically unhealthy and obese, and whether genetics and lifestyle factors play a role in both development and reversal of such. Likewise, studies conducted in aging cohorts would give insight into the prevalence of this phenotype in the elderly, clarifying the time-dependent relationship of obesity and metabolic abnormality presentation.

## Conclusion

MHO is an important, emerging phenotype with risks somewhere intermediate between healthy, normal weight and unhealthy, obese individuals. Successful work towards a universally accepted definition of MHO would improve (and simplify) future studies and strengthen inter-study comparisons. Usefulness of a definition inclusive of insulin sensitivity in concert with stricter criteria for metabolic syndrome components should be explored, as well as the potential inclusion of fatty liver. As obesity continues to rise, this phenotype will become increasingly important. Clinicians exercise caution in reassuring patients that the metabolically benign phenotype is safe, as increased risk of cardiovascular events and death remains a possibility based on the limited data reviewed.

## Abbreviations

ABI: Ankle-brachial index; AC: Aortic calcium; aPWV: Aortic pulse wave velocity; BMI: Body mass index; CAC: Coronary artery calcium; CCA-IMT: Common carotid artery intima media thickness; CRP: C-reactive protein; E/A: Early/Late; IR: Insulin resistance; IS: Insulin sensitive; LVMI: Left ventricular mass index; MetS: Metabolic syndrome based on the NCEP ATP III Definition: waist circumference ≥ 102 cm (male), ≥ 88 cm (female), TG ≥ 150 mg/dL or use of lipid lowering medications HDL-C < 40 mg/dL (male) < 50 mg/dL (female) or use of lipid lowering medications, blood pressure ≥ 130/85 mmHg or use of antihypertensive medications fasting plasma glucose ≥ 110 mg/dl or use of antidiabetic medications.; MHNW: Metabolically healthy, normal weight; MHO: Metabolically healthy obese; MUHO: Metabolically unhealthy obese; MUNW: Metabolically unhealthy, normal weight; NAFLD: Non-alcoholic fatty liver disease; NR: Not reported; VI: Vascular inflammation; WBC: White blood cell.

## Competing interests

The authors have no competing interests. Please send reprint requests to Khurramn@baptisthealth.net

## Authors’ contributions

LR and EA carried out the literature review and data extraction. LR, WM, MB(Blaha), RD, and AS participated in table construction. LR, AA, TF, and MA drafted the manuscript. RB and MB(Budoff) contributed to subclinical CVD section. MR and TT assisted with discussion section. KN conceived of the manuscript concept and oversaw all aspects of manuscript preparation. All authors read and approved the final manuscript.

## Pre-publication history

The pre-publication history for this paper can be accessed here:

http://www.biomedcentral.com/1471-2458/14/14/prepub
